# Characterization of Inulin-Type Fructan from *Platycodon grandiflorus* and Study on Its Prebiotic and Immunomodulating Activity

**DOI:** 10.3390/molecules24071199

**Published:** 2019-03-27

**Authors:** De-Jiang Pang, Chao Huang, Mei-Ling Chen, Yu-Long Chen, Yu-Ping Fu, Berit Smestad Paulsen, Frode Rise, Bing-Zhao Zhang, Zheng-Li Chen, Ren-Yong Jia, Li-Xia Li, Xu Song, Bin Feng, Xue-Qin Ni, Zhong-Qiong Yin, Yuan-Feng Zou

**Affiliations:** 1 Natural Medicine Research Center, College of Veterinary Medicine, Sichuan Agricultural University, Chengdu 611130, China; dejiangpang@163.com (D.-J.P.); huangchao@sicau.edu.cn (C.H.); morningyeah2@163.com (M.-L.C.); yupingfu424@163.com (Y.-P.F.); lilixia905@163.com (L.-X.L.); songx@sicau.edu.cn (X.S.); yinzhongq@163.com (Z.-Q.Y.); 2 Key Laboratory of Animal Disease and Human Health of Sichuan Province, College of Veterinary Medicine, Sichuan Agricultural University, Chengdu 611130, China; chzhli75@163.com (Z.-L.C.); jiary@sicau.edu.cn (R.-Y.J.); 3 Sichuan Academy of Forestry, Chengdu 610081, China; 4 Department of Pharmaceutical Chemistry, School of Pharmacy, University of Oslo, P.O. Box 1068, Blindern, 0316 Oslo, Norway; b.s.paulsen@farmasi.uio.no; 5 Department of Chemistry, University of Oslo, P.O. Box 1033, Blindern, 0315 Oslo, Norway; frode.rise@kjemi.uio.no; 6 Shenzhen Institutes of Advanced Technology, Chinese Academy of Science, Shenzhen 518055, China; bz.zhang@siat.ac.cn; 7 Animal Nutrition Institute, Sichuan Agricultural University, Chengdu 611130, China; fengbin@sicau.edu.cn; 8 Animal Microecology Institute, College of Veterinary Medicine, Sichuan Agricultural University, Chengdu 611130, China; xueqinni@foxmail.com

**Keywords:** *Platycodon grandiflorus*, inulin-type fructan, prebiotic activity, immunomodulation

## Abstract

*Platycodon grandiflorus* is a plant widely used in traditional Chinese medicine, of which polysaccharides are reported to be the main components responsible for its bio-functions. In this work, the inulin-type fructan (PGF) was obtained by DEAE anion exchange chromatography from the water extracted from *P. grandifloras*. Characterization was performed with methanolysis, methylation, and NMR and the results showed that PGF is a β-(2-1) linked fructan, with terminal glucose and with a degree of polymerization of 2–10. In order to study its biofunctions, the prebiotic and immunomodulation properties were assayed. We found that PGF exhibited good prebiotic activity, as shown by a promotion on six strains of lactobacillus proliferation. Additionally, the PGF also displayed direct immunomodulation on intestinal epithelial cells and stimulated the expressions of anti-inflammatory factors. These results indicated that the inulin from *P. grandiflorus* is a potential natural source of prebiotics as well as a potential intestinal immunomodulator, which will be valuable for further studies and new applications.

## 1. Introduction

The immune system is responsible for the initiation of immune response and is of critical importance for the recognition or elimination of foreign substances to keep an immune balance [1]. The gastrointestinal tract is the interface between host and environment and it acts as the largest immune organ of human beings [2]. Numerous commensal bacteria are housed in the gastrointestinal tract and are thus implicated in the modification of the immune response in the gastrointestinal tract [3,4,5]. Among these commensal bacteria, probiotics, which are defined as ‘live microorganisms that confer a health benefit on the host’, are, with large evidence, emerging as important participants in promoting a non-immunologic gut defense barrier and improving the intestine’s immunological barrier [6,7]. Therefore, probiotics are widely used in gastrointestinal disorders, especially in intestinal inflammation resulting from imbalance of the intestinal microflora. Oral probiotics are the most used method, but defects and limitations of these have elicited interest in exploring biomolecules with prebiotic activity to modulate the intestinal immune response.

Several polysaccharides, especially the neutral ones, were reported to function as prebiotics. The inulin-type fructan, which is widely distributed in medical plants and is most abundant in *Compositae* and in *Platycodon grandifloras* [8,9], is one of the neutral types of polysaccharides. As a natural storage polysaccharide with a variety of prospects in food and pharmaceutical applications, inulin is a linear plant oligo- and poly-saccharide, which consists of a minimum two fructose-units and at least one β-(2-1) fructosyl-fructose glycosydic bond [10,11]. A variety of bio-functions have been demonstrated for inulin, including as an anti-oxidant [12,13], immune and metabolism regulation [9,11,14,15], and for intestinal microbial balance improvement [16]. A beneficial effect of inulin was reported to selectively stimulate the growth and/or activity of one or a limited number of bacteria in the colon [17]. Inulin from different plants, such as *Burdock* [18], *Stevia rebaudiana* [19], and *Codonopsis pilosula* [20], showed prebiotic activity on lactobacilli in vitro. In addition to functioning as a prebiotic to indirectly modulate intestinal immune response, inulin could be detected by gut dendritic cells or intestinal epithelial cells and directly stimulate the anti-inflammatory cytokines release implicated in immune response [21,22,23]. Therefore, a great prospective application of inulin could be predicted for the treatment of diseases related to intestinal microbial imbalance or immune dysfunction.

Platycodonis Radix, the root of *Platycodon grandiflorus* (Jacq.) A. DC., is a traditional herb widely used for the treatment for sore throats, excessive phlegm, and coughing in Eastern Asia. Modern pharmacology studies have further expanded its applications for anti-asthmatic, hepatoprotective, anti-oxidant, immunoregulation, and anti-tumor effects [24,25]. As both an edible and a pharmaceutical material, *P. grandiflorus* not only contains nutrient substances, such as amino acids, saponins, flavonoids, anthocyanins, and phenolics, but is also rich in polysaccharides [25,26]. Previous studies have demonstrated that the polysaccharide is one of the major active ingredients of *P. grandiflorus* and the function studies of them have displayed bio-activities, including as an anti-oxidative [27], for anti-angiogenesis [28], and especially for immunoregulation [29]. For example, *P. grandiflorus* polysaccharides were reported to induce the maturation of dendritic cells or macrophage, accompanied with increased expressions of MHC-I/II, co-stimulatory molecules, and cytokines [29,30]. However, the composition and structure of these polysaccharides, including the inulin-type fructan, have not been clearly identified and characterized. The neutral polysaccharides, but not inulin, from *P. grandiflorus* were isolated by DEAE-cellulose chromatography before, but its immunomodulating bioactivity and prebiotic activity are not well defined for now [28,31].

In this study, we aim to isolate a fructan from *P. grandifloras* using water extraction and ethanol precipitation, followed by ion exchange chromatography, to further characterize its structure and investigate its potential prebiotic and immunomodulating activity in vitro.

## 2. Results and Discussions

### 2.1. Purification of Inulin from P. grandiflorus

The crude polysaccharide from *P. grandiflorus* (PG) was obtained by using water extraction and ethanol precipitation, which is different from other studies, as they used a 70% ethanol extraction [32]. The crude polysaccharide PG (1.2 g) was dissolved in distilled water and subjected to DEAE ion exchange chromatography six times (200 mg for each time). The distilled water elutes (PGF) were collected and combined, concentrated, and lyophilized, with a yield of 59.4% (0.7127 g PGF from 1.2 g PG). Oka et al. purified several inulin-type fructans from *P. grandifloras*, with different degrees of polymerization, using ion exchange chromatography, but they used a different elution buffer and more steps for purification [32].

### 2.2. Chemical Characterization of PGF

The results of methanolysis and the Urea-HCl colorimetric method indicated that PGF only contains glucose (Glc) and fructose (Fru). The results of a Folin–Ciocalteu assay and a Bio-Rad protein assay indicated that there were no phenolic compounds and trace amount of protein was detected in PGF. The inter-glycosidic linkages were identified by GC-MS, after methylation analysis, and the results showed that PGF was composed of mainly terminal-Fru*f*, terminal-Glc*p*, and 2, 1-linked Fru*f*, with a molar ratio of 1, 2, and 7, respectively.

**Figure 1:**
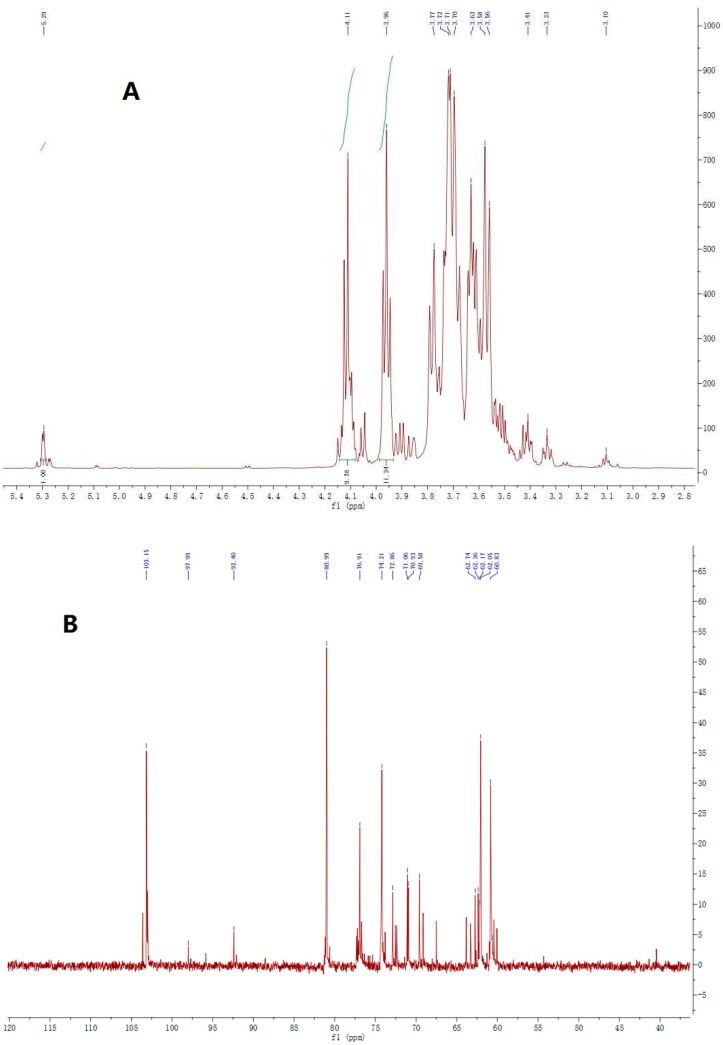
The^1^H-NMR (**A**) and ^13^C-NMR (**B**) spectrum of PGF.

The NMR signals were characterized and compared with chemical shift values from literature [20,32,33,34]. The ^1^H NMR spectrum of PGF contained a main anomeric proton at 5.29 ppm, belonging to the H1 of terminal linked Glc*p* (Figure 1A). The other signals at δ 4.11 and 3.96 corresponded to the H3-Fru*f* and H4-Fru*f*, respectively. The degree of polymerization (DP) was calculated by the mean ratio between the integral proton signal (H3-Fru*f* and H4-Fru*f*) and the integral of the Glc*p* signal (H^1^-Glc*p*) [35], with the result suggesting the DP of PGF was about 10. The DP of PGF, obtained in the present study, is similar to those extracted from *P. grandiflorus* using a 70% ethanol extraction method, in which the DP ranged from 2 to 9 [32]. The signals between δ 3.56–3.77 corresponded to H1-Fru*f*, H5-Fru*f*, and H6-Fru*f* (Figure 1A). The ^13^C NMR spectrum contained six major signals, one major signal at 103.15 ppm, assigned to the C2 carbon of Fru*f*, and five signals at 60.83, 76.91, 74.21, 80.99, and 62.05 ppm, as the signals of C1-Fru*f*, C3-Fru*f*, C4-Fru*f*, C5-Fru*f*, and C6-Fru*f*, respectively (Figure 1B). The carbon atom signal at 92.39 ppm was assigned to Glc*p* because signals beyond 100 ppm pointed to ketose residues. The other signals between 60.42–71.06 ppm belonged to the C2–C6 of the Glc*p* (Figure 1B). All these results indicated that the PGF was linked on C2 of Fru*f* and C1 of the terminal-Glc*p*, with a backbone of (2→1)-Fru*f*, identified as a typical inulin-type fructan, as follows: A β-(2→1)-linked configuration at the anomeric carbon of fructosyl residues, connected with terminal Glc*p* residue by an α-d-(1→2) bond, combing the results of monosaccharide compositions and glycosidic linkage. Those results indicated the structure of PGF as α-d-Glc*p*-(1→2)–(β-d-Fru*f*-(2→1)-β-d-Fruf)n-(2→1)-β-d-Fru*f*, an inulin-type fructan.

### 2.3. Prebiotic Activity of PGF

The inulin-type fructan cannot be hydrolyzed or absorbed in the upper part of the gastrointestinal tract (GIT), but is degraded by the gut microbiota in the large intestine through processes of fermentation [9,11]. Inulin fermentation will improve the composition of the gut microbiota and is beneficial for the probiotic’s enrichment. Additionally, short-chain fatty acids (SCFAs) will be produced during fermentation and implicated in multiple cellular and physiological processes, such as energy metabolism, hormone release, and immune response [36,37]. In our study, PGF (DPn 2–10) was separately fermented with six strains of lactobacilli and significantly higher bacterial optical densities were detected in the medium with PGF than that in the basal medium (without sugar) (Table 1). In this study, two available commercial prebiotics, P95s (DPn 2–9) and Orafti^®®^ HP (DPav ≥ 23), were selected to compare with PGF. The bacterial optical densities in the medium containing Orafti^®®^ HP were almost the same as in the basal medium, suggesting that Orafti^®®^ HP displayed no prebiotic effect on all the lactobacilli. Differently, the bacterial optical density in the medium containing P95s was dramatically increased. Among them, except for the *L. gasseri* KQ11-1, the optical density of the other bacteria was higher than that in the PGF group. These results demonstrated that the activity of probiotics on lactobacilli was related to its DP, which was consistent with previous reports [19,35]. Similarly, besides *L. rhamnosus* LGG, the pH values of the PGF medium, after incubation with other strains of lactobacilli, were lower than the basal medium and were the lowest in P95s group. Additionally, of course, the pH was changed with the species of lactobacilli (Table 2). The reductions of pH in the PGF or P95s groups were probably due to the metabolites, like lactic acid and acetic acid, produced by lactobacilli, or the SCFAs, the fermentation products from inulin [11,38]. All these results indicated that the PGF was a potential prebiotic, stimulating the growth of lactobacilli and with a lower pH. 

As shown above, the PGF displayed prebiotic activity on all the strains of lactobacilli, but with unequal effects. The bacterial optical density of BSGP201683 in the medium with PGF increased 3-fold compared to the basal medium, but that of KQ11-1 was just 1.2-fold. The difference in inulin utilization between bacteria may be due to their enzymatic equipment, especially the hydrolases and transportases, which are responsible for the hydrolysis of fructan in position β-(2→1) [11,38].

### 2.4. PGF Promote the Anti-Inflammatory Factors Release of IPEC-J2 Cells

**Figure 2:**
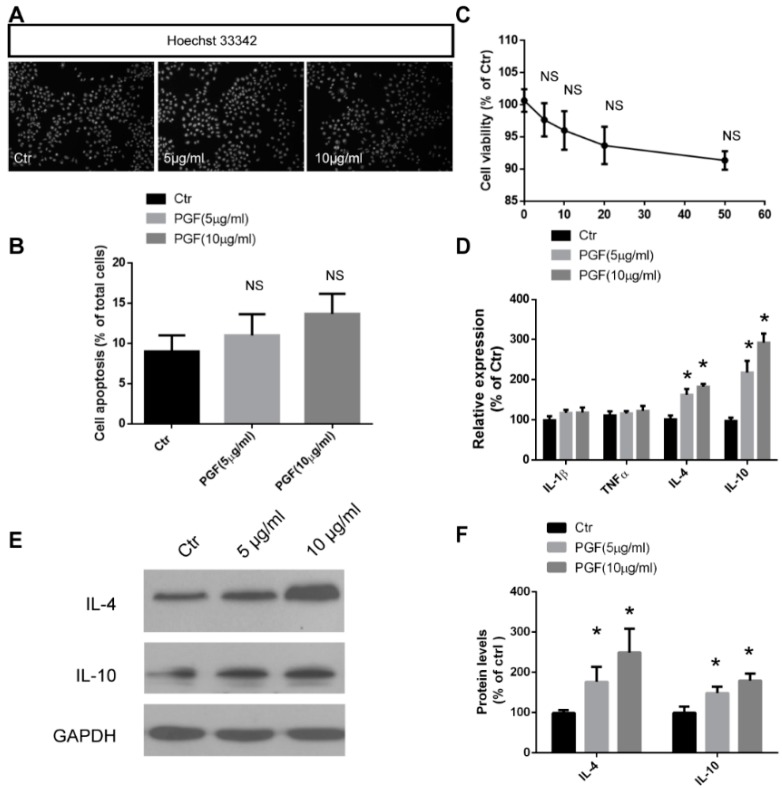
PGF promote the anti-inflammatory factors’ release of IPEC-J2 cells. (**A**) Representative images display the nuclear morphology of IPEC-J2 cells under different doses of PGF treatment for 24 h, labeled with Hoechst 33342. (**B**) Quantification shows that PGF doesn’t induce cell apoptosis in IPEC-J2 cells, according to Hoechst staining. (**C**) Quantification shows the cell viability of IPEC-J2 cells treated with different concentrations of PGF for 24 h. (**D**) Quantification shows increased expression levels of anti-inflammatory factors, but not pro-inflammatory factors, after the treatment of PGF (5 μg/mL and 10 μg/mL). (**E** and **F**) Western blots and quantification show increased protein levels of anti-inflammatory factors after the treatment of PGF (5 μg/mL and 10 μg/mL). Error bars indicate SEM. * *p* < 0.05, NS, not significant.

Recent studies have noticed that prebiotic carbohydrates, such as inulin, may elicit additional direct effects on immunomodulation [1,21]. This may occur via direct contact between inulin and intestinal epithelial cells, to modify epithelial tight junction integrity, or the release of pro- or anti-inflammatory factors [39]. To evaluate the immunomodulating effects of PGF on intestinal epithelial cells, in vitro assays were performed with a porcine jejunum epithelial cell line (IPEC-J2). Firstly, the cytotoxicity of the PGF was evaluated and we found that the additional PGF exhibited no cellular toxicity up to 50 μg/mL, which was indicated by CCK-8 assays and Hoechst 33342 staining (Figure 2A–C). Then, the expressions of pro- or anti-inflammatory factors were detected in IPEC-J2 cells after PGF treatment. It was found that anti-inflammatory factor (IL-4 and IL-10) mRNA levels were significantly increased in a dose dependent manner, but little pro-inflammatory change (IL-1β and TNF-α) was present (Figure 2D). Additionally, protein levels of IL-4 and IL-10 were further evaluated by western blots, which found the same results as with mRNA levels (Figure 2E,F). These data demonstrated that, except functioning as probiotics, PGF may be implicated in the intestinal immune response directly through an activation of the anti-inflammatory factor’s expression or even release. However, the exact mechanism behind this effect is not well understood and needs to be further studied. It is suggested that pathogen recognition receptors (PRRs), such as toll-like receptors (TLRs), C-type lectin receptors, NOD-like receptors (NLRs), and RIG-I-like receptors (RLRs), possess carbohydrate binding properties and are involved in the perception of carbohydrates in the intestine [40,41,42]. When carbohydrates ligate with PRRs, cellular signals are elicited and further modulate intestinal barrier function and immune response. It is also reported that inulin-type fructans may even be inserted into the lipid membrane and subsequent signal transduction may be induced [43,44,45]. Therefore, more studies are needed to clarify whether PGF could bind to some PRRs, or insert into the lipid membrane to directly stimulate the intestinal immune response.

## 3. Materials and Methods

### 3.1. Materials and Reagents

The roots of *P*. *grandiflorus* were purchased from the Lotus Pond Chinese herbal medicine market, and identified as *Platycodon grandiflorus* (Jacq.) A. DC. by Dr. Yuan-Feng Zou of Sichuan Agricultural University. The roots were dried in a drying oven (DHG-9420A, Yi-heng Technology Co., Ltd., Shanghai, China) at 40 °C overnight and pulverized to a fine powder by a mechanical grinder, then passed through 0.25 mm mesh.

The MRS medium (HB0384-1), peptone (HB8276), and tryptone (HB8270) were purchased from Hopebio Biotechnology Co., Ltd. (Qingdao, China); the yeast extract powder (JM-500) was purchased from Biotopped Science and Technology Co., Ltd. (Beijing, China); the McIntosh Turbidimetric tube (G60346) was obtained from Wenzhou Kangtai Biotechnology Co., Ltd. (Wenzhou, Zhejiang, China); and the standards fructo-oligosaccharide (QHT-FOS-P95S) and inulin (Orafti^®®^ HP) were purchased from Quantum Hi-Tech Biological Co., Ltd. (Jiangmen, China) and Beneo-Orafti (Oreye, Belgium), respectively.

The standards of fructose (Fru) and glucose (Glc) were purchased from Solarbio (Beijing, China). All other chemicals, such as phenol, sulfuric acid, acetone, boric acid, glycerin, etc., were of analytical grade, obtained from the Chengdu Kelong chemical factory (Chengdu, China).

### 3.2. Isolation of Inulin from P. grandiflorus

Seventy grams of *P. grandiflorus* powder were extracted by refluxing petroleum ether to remove low molecular weight and lipid soluble compounds. The residue was dried and extracted three times, with distilled water (water/material, 30 mL/1 g) for 2 h each time. These extracts were filtered and concentrated, then precipitated with 3-fold volumes of ethanol at 4 °C for 24 h. After centrifugation at 1000 rpm for 15 min, the precipitate was freeze-dried, named PG (the crude polysaccharides from *P. grandiflorus*).

The crude polysaccharide PG (1.2 g) was dissolved in distilled water and then centrifuged (1000 rpm, 10 min) to remove insoluble impurities. The supernatant was filtered through a 0.45 μm filter for purification. These PG solutions were applied to a DEAE-Sepharose (Fast Flow, FF) column (50 mm × 40 cm, Beijing Rui Da Heng Hui Science Technology Development Co., Ltd., Beijing, China), and eluted with distilled water. The flow rate was 2.0 mL/min. It was collected until no sugar was detected by the PABR method (Boratyński, 1984). Then, the eluate was concentrated and lyophilized and named PGF.

### 3.3. Characterization of Inulin from P. grandiflorus

The monosaccharide composition of PGF was determined using methanolysis. Approximately 1 mg of PGF was dissolved in 3 M hydrochloric acids in anhydrous methanol for 24 h at 80 °C and then derivatized by trimethylchlorsilane (TMS). The derivatives were analyzed using gas chromatography [46,47]. Mannitol was added to the samples as the internal standard. The presence of fructose was tested with the Urea-HCl colorimetric method [48].

The total amount of phenolic compounds in the purified polysaccharide fractions were quantitatively determined using the Folin–Ciocalteu assay [49]. The protein content of the polysaccharide fractions was determined by the Bio-Rad protein assay, based on the method of Bradford [50].

The glycisidic linkages were determined by methylation. The derivatives were analyzed by a GCMS-QP2010 (Shimadzu, Kyoto, Japan). The relative amount of each type of linkage was determined based on the area of each compound and related to the monosaccharide composition of each compound [47].

Approximately 20 mg of PGF was first deuterium exchanged three times by freeze-drying in D_2_O (10 mg/mL). The ^1^H NMR and ^13^C NMR spectra of PGF were recorded on a Bruker AV600 instrument (Bruker, Rheinstetten, Germany) at 25 °C.

### 3.4. Prebiotic Activity

#### 3.4.1. Bacterial Strains

Six strains of lactobacilli were used to determine the prebiotic activity in vitro. The *L. johnsonii* (BS15, CCTCC: M2013663), *L. plantarum* (BS10, CCTCC: M2012487), *L. plantarum* (BSGP201683, CCTCC: M2016425), *L. rhamnosus* GG (LGG, ATCC53103) and *Weissella confusa* X3 were gifts from professor Xue-Qin Ni of Animal Microecology Institute, College of Veterinary Medicine, Sichuan Agricultural University, China. *L. gasseri* (KQ11-1, ATCC 33323) was a gift from Dr. Bing-Zhao Zhang of Shenzhen Institutes of Advanced Technology, Chinese Academy of Science, China. They were stored at −80 °C in the MRS medium, with 20% glycerin.

#### 3.4.2. Bacterial Growth

The basal medium (10 g tryptone, 10 g peptone, 5 g yeast extract, 1 mL of Tween 80, 0.5 g/L-cysteine hydrochloride, 1 g/L carbohydrate source, and 1 L of distilled water, pH 6.5) and the MRS medium were autoclaved at 121 °C for 20 min. The PGF (10 mg/mL in saline, the same below), P95s, Orafti^®®^ HP, and glucose were used as carbon sources after being filtered through a sterile filter (0.22 μm), in which the P95s (96.1% fructo-oligosaccharides, DPn 2–9, with 2.7% glucose, fructose, and sucrose, product of the partial enzymatic hydrolysis of chicory inulin) and Orafti^®®^ HP (99.8% inulin, DPav ≥ 23, with 0.2% glucose, fructose, and sucrose), commercially available prebiotics, were used for comparison with PGF. Meanwhile, the basal medium, without carbohydrates, was used as the negative control [35,51].

These six strains of lactobacilli were incubated in 50 mL of the MRS medium at 37 °C overnight in an anaerobic chamber (Thermo Scientific 1029, in 85% N_2_, 10% H_2_, 5% CO_2_), then centrifuged (2129 g, 10 min) and resuspended in the saline and basal mediums to remove the carbon source. Finally, they were resuspended with the basal medium containing these four different carbon sources above (PGF, P95s, Orafti^®®^ HP and glucose) at a concentration of 10^7^–10^8^ CFU/mL bacteria [38], adjusted by a McIntosh Turbidimetric tube. The 5 milliliter bacterial suspensions were divided into test tubes and then incubated at 37 °C for 0 h and 24 h. All the test tubes were set in triplicate. After 0 h and 24 h of incubation, 200 μL of the basal medium was added to the 96-well plates and the densities of the bacteria were measured at the wave-length of 600 nm (A_600_) by a Multiscan Spectrum (Thermo Scientific, Varioskan Flash, Waltham, MA, USA). The bacterial growth was presented as the increment in A_600_ (ΔA_600_) during 24 h of incubation in anaerobic chamber. The pH was measured by a pH meter (A115200, Lichen Instrument Technology Co. Ltd., Changsha, Hunan, China) after removing bacteria by centrifuge, at 2231g for 20 min after 24 h of incubation. Each tube was tested three times to ensure high accuracy and precision.

### 3.5. Cell Culture

The IPEC-J2 was obtained from the Shanghai Institutes of Biological Sciences, Chinese Academy of Sciences (Shanghai, China). IPEC-J2 cells were cultured in Dulbecco’s modified Eagle’s medium (DMEM; HyClone, GE Healthcare Life Sciences, Logan, UT, USA), supplemented with 10% fetal bovine serum (FBS; Gibco; Thermo Fisher Scientific, Inc., Waltham, MA, USA) and 1% penicillin–streptomycin (Invitrogen; Thermo Fisher Scientific, Inc., Waltham, MA, USA). Cells were maintained at 37 °C in a humidified atmosphere (5% CO_2_).

### 3.6. Cell Viability Assay

Cell viability was determined by CCK-8 assay. The cells were seeded with a density of 5 × 10^3^ cells/well in 96-well plates and incubated, at 37 °C, in a complete medium (DMEM with 10% FBS), with serial dilutions of PGF, for 24 h. CCK-8 was added to each well of the 96-well plate (CCK-8, plus fresh medium at a fixed ratio of 1:10, 100 μL/well), followed by further incubation for 1 h. The absorbance was measured at 450 nm using a microplate reader (Thermo Fisher Scientific, Inc.).

### 3.7. Hoechst 33342 Staining

Briefly, IPEC-J2 were plated in 12-well plates with a density of 5 × 10^4^ cells/well and treated with PGF at 37 °C for 24 h. The cells were washed in PBS three times and incubated in the Hoechst 33342 solution (10 μg/mL) for 30min at 4 °C. Finally, fluorescence microscopy was performed to observe the nuclear changes of IPEC-J2 cells. For each treatment group, ≥1000 cells were analyzed in triplicate.

### 3.8. Quantitative Realtime PCR

RNA extraction from IPEC-J2 cells and real time PCR for anti- or pro-inflammatory factor detection was performed as previously reported [52]. Briefly, IPEC-J2 cells were lysed with Trizol Regent (Invitrogen, Waltham, MA, USA) and the total RNA was extracted from the IPEC-J2 cells, according to the manufacturer’s instructions. The quality of RNA was assessed by agarose gel and the concentration was measured with a spectrophotometer (NanoDrop 2000, Thermo Scientific, Shanghai, China). The total RNA was subjected to reverse transcription with a reverse transcriptase, according to the manufacturer’s instructions (Fermentas, Waltham, MA, USA). Quantitative real-time PCR was performed using the Bio-Rad CFX96 system and the relative gene expression was normalized to the internal control actin. Primer sequences for SYBR Green probes of the target genes are described in Table 3.

### 3.9. Statistical Analysis

All data obtained above were analyzed by SPSS statistics software (Version 20.0, USA), including the one-way analysis of variance with Duncan’s test, presented as the mean ± standard deviation (SD). The differences between the groups were evaluated at the statistically significant level of *p* < 0.05.

## 4. Conclusions

In summary, a purified inulin-type fructan from *P. grandiflorus* was obtained by DEAE ion exchange chromatography. Structural analysis by methanolysis, methylation, and NMR identified PGF as a typical inulin-type fructan having (2-1) linkages. Moreover, this PGF was shown to be an effective prebiotic on lactobacilli and an immunomodulator on intestinal epithelial cells. However, more in vivo studies are needed prior to the further advancement of PGF applications.

## Figures and Tables

**Table 1:** Capacity to ferment PGF and commercial prebiotics by lactobacilli.

Groups	The Bacterial Density (△A_600,_ n = 4)				
	PGF	Orafti^®®^ HP	P95s	Basal Medium	Glucose
*L. Johnsonii* BS15	0.037 ± 0.001 ^a,b,A^	0.015 ± 0.001 ^a,A^	0.051 ± 0.033 ^b,A^	0.016 ± 0.002 ^a,A^	0.060 ± 0.003 ^b,A^
*L. plantarum* BS10	0.056 ± 0.001 ^a,A^	0.030 ± 0.001 ^b,B^	0.198 ± 0.006 ^c,B^	0.031 ± 0.003 ^b,B^	0.177 ± 0.00 ^d,B^
*plantarum* BSGP201683	0.12 ± 0.025 ^a,B^	0.04 ± 0.001 ^b,C^	0.24 ± 0.002 ^c,C^	0.04 ± 0.003 ^b,C^	0.39 ± 0.005 ^d,C^
*L. rhamnosus* LGG	0.045 ± 0.002 ^a,A^	0.027 ± 0.005 ^b,B^	0.031 ± 0.002 ^b,A^	0.027 ± 0.004 ^b,B^	0.196 ± 0.007 ^c,B^
*L. gasseri* KQ11-1	0.104 ± 0.004 ^a,B,C^	0.088 ± 0.001 ^b,D^	0.091 ± 0.002 ^b,D^	0.084 ± 0.002 ^b,D^	0.324 ± 0.014 ^c,D^
*Weissella confusa* X3	0.085 ± 0.005 ^a,C^	0.040 ± 0.002 ^b,E^	0.224 ± 0.004 ^c,C^	0.037 ± 0.002 ^b,E^	0.395 ± 0.026 ^d,C^

Notes: Data are expressed as the increase in A_600_ of the bacterial suspension with 24 h incubations; values are means from triplicate determination ± standard deviation. ^a–d^ Data in lines with different superscripts are significantly (*p* < 0.05). ^A–E^ Data in columns with different superscripts are significantly (*p* < 0.05).

**Table 2:** The final pH of mediums containing PGF and commercial prebiotics by lactobacilli.

Groups	pH (n = 4)				
	PGF	Orafti^®®^ HP	P95s	Basal Medium	Glucose
*L. Johnsonii* BS15	5.87 ± 0.15 ^a,A,B^	6.03 + 0.06 ^b,A^	5.67 + 0.05 ^c,A^	6.10 + 0.00 ^b,A^	5.03 + 0.06 ^d,A^
*L. plantarum* BS10	5.93 ± 0.02 ^a,A^	6.08 ± 0.01 ^b,A^	5.49 ± 0.01 ^c,B^	6.08 ± 0.02 ^b,A^	5.03 ± 0.01 ^d,A^
*L. plantarum* BSGP201683	5.54 ± 0.03 ^a,C^	5.68 ± 0.02 ^b,B^	5.37 ± 0.02 ^c,C^	5.78 ± 0.02 ^d,B^	5.06 ± 0.01,^e,A^
*L. rhamnosus* LGG	5.76 ± 0.06 ^a,B,D^	5.82 ± 0.12 ^a,C^	5.82 ± 0.06 ^a,D^	5.89 ± 0.05 ^a,C^	4.64 ± 0.05 ^b,B^
*L. gasseri* KQ11-1	5.40 ± 0.05 ^a,E^	5.51 ± 0.01 ^a,D^	5.50 ± 0.09 ^a,B^	5.48 ± 0.09 ^a,D^	4.45 ± 0.07 ^b,C^
*Weissella confusa* X3	5.70 ± 0.00 ^a,D^	5.80 ± 0.00 ^b,C^	5.40 ± 0.00 ^c,C^	5.80 ± 0.00 ^b,B^	5.07 ± 0.06 ^d,A^

Notes: Data are expressed as the final pH of the medium after 24 h incubations; values are means from triplicate determination ± standard deviation. ^a–d^ Data in lines with different superscripts are significantly (*p* < 0.05). ^A–E^ Data in columns with different superscripts are significantly (*p* < 0.05).

**Table 3:** Primer sequences for qRT-PCR.

Gene	Primers
IL-1β	Fr 5′-GGCCGCCAAGATATAACTGA-3′
	Rv 5′-GGACCTCTGGGTATGGCTTTC-3′
TNF-α	Fr 5′-CGCCCACGTTGTAGCCAATGT-3′
	Rv 5′-CAGATAGTCGGGCAGGTTGATCTC-3′
IL-4	Fr 5′-TACCAGCAACTTCGTCCAC-3′
	Rv 5′-ATCGTCTTTAGCCTTTCCAA-3′
IL-10	Fr 5′-AACAAGAGCAAGGCCGTG-3′
	Rv 5′-AATAGTTCACAGAGAGGCTCGG-3′
β-Actin	Fr 5′-GTACCACTGGCATTGTGATG-3′
	Rv 5′-ATCTTCATGGTGCTAGGAGC-3′

## References

[1] Kober M.M., Bowe W.P. (2015). The effect of probiotics on immune regulation, acne, and photoaging. Int. J. Womens Dermatol..

[2] Mowat A.M., Viney J.L. (2010). The anatomical basis of intestinal immunity. Immunol. Rev..

[3] Bischoff S.C., KräMer S. (2010). Human mast cells, bacteria, and intestinal immunity. Immunol. Rev..

[4] Hill D.A., Artis D. (2010). Intestinal Bacteria and the Regulation of Immune Cell Homeostasis. Annu. Rev. Immunol..

[5] Kamada N., Seo S.U., Chen G.Y., Nunez G. (2013). Role of the gut microbiota in immunity and inflammatory disease. Nat. Rev. Immunol..

[6] Bron P.A., Kleerebezem M., Brummer R.-J., Cani P.D., Mercenier A., MacDonald T.T., Garcia-Ródenas C.L., Wells J.M. (2017). Can probiotics modulate human disease by impacting intestinal barrier function?. Br. J. Nutr..

[7] Borchers A.T., Selmi C., Meyers F.J., Keen C.L., Gershwin M.E. (2009). Probiotics and immunity. J. Gastroenterol..

[8] Apolinario A.C., de Lima Damasceno B.P.G., de Macedo Beltrao N.E., Pessoa A., Converti A., da Silva J.A. (2014). Inulin-type fructans: A review on different aspects of biochemical and pharmaceutical technology. Carbohydr. Polym..

[9] Shoaib M., Shehzad A., Omar M., Rakha A., Raza H., Sharif H.R., Shakeel A., Ansari A., Niazi S. (2016). Inulin: Properties, health benefits and food applications. Carbohydr. Polym..

[10] Kelly G. (2008). Inulin-type prebiotics—A review: Part I. Altern. Med. Rev..

[11] Wilson B., Whelan K. (2017). Prebiotic inulin-type fructans and galacto-oligosaccharides: Definition, specificity, function, and application in gastrointestinal disorders. J. Gastroenterol. Hepatol..

[12] Wang Y.-X., Zhou J.-D., Cao F., Yu Z.-L., Zhang F.-R., Huang R.-X. (2011). Extraction, Purification and Polymerization Degree Distribution of Inulin from *Jerusalem artichoke* Grown in Saline-alkaline Soil. Food Sci..

[13] Liu D., Ping W.U. (2015). Studies on the Antioxidant Activity of Inulin and Its Mechanism. J. Food Sci. Biotechnol..

[14] Salazar N., Dewulf E.M., Neyrinck A.M., Bindels L.B., Cani P.D., Mahillon J., de Vos W.M., Thissen J.-P., Gueimonde M., Clara G. (2015). Inulin-type fructans modulate intestinal Bifidobacterium species populations and decrease fecal short-chain fatty acids in obese women. Clin. Nutr..

[15] Xiong Z.W., Dong Q., Wang Q. (2015). Effect of inulin bread on blood glucose and lipid of mice with diabetes induced by STZ. Sci. Technol. Food Ind..

[16] Xu X., Xu P., Ma C., Tang J., Zhang X. (2013). Gut microbiota, host health, and polysaccharides. Biotechnol. Adv..

[17] Morris C., Morris G.A. (2012). The effect of inulin and fructo-oligosaccharide supplementation on the textural, rheological and sensory properties of bread and their role in weight management: A review. Food Chem..

[18] Du Y.-J., Xie C.-P. (2011). The Inulin Extraction from Burdock and Bacteriostasis Research. Food Res. Dev..

[19] Lopes S.M.S., Francisco M.G., Higashi B., de Almeida R.T.R., Krausová G., Pilau E.J., Gonçalves J.E., Gonçalves R.A.C., de Oliveira A.J.B. (2016). Chemical characterization and prebiotic activity of fructo-oligosaccharides from *Stevia rebaudiana* (Bertoni) roots and in vitro adventitious root cultures. Carbohydr. Polym..

[20] Fu Y.-P., Li L.-X., Zhang B.-Z., Paulsen B.S., Yin Z.-Q., Huang C., Feng B., Chen X.-F., Jia R.-R., Song X. (2018). Characterization and prebiotic activity in vitro of inulin-type fructan from *Codonopsis pilosula* roots. Carbohydr. Polym..

[21] Vogt L., Meyer D., Pullens G., Faas M., Smelt M., Venema K., Ramasamy U., Schols H.A., De Vos P. (2015). Immunological properties of inulin-type fructans. Crit. Rev. Food Sci. Nutr..

[22] Hapfelmeier S., Müller A.J., Stecher B., Kaiser P., Barthel M., Endt K., Eberhard M., Robbiani R., Jacobi C.A., Heikenwalder M. (2008). Microbe sampling by mucosal dendritic cells is a discrete, MyD88-independent stepin ΔinvG S. Typhimurium colitis. J. Exp. Med..

[23] Eiwegger T., Stahl B., Haidl P., Schmitt J., Boehm G., Dehlink E., Urbanek R., Szépfalusi Z. (2010). Prebiotic oligosaccharides: In vitro evidence for gastrointestinal epithelial transfer and immunomodulatory properties. Pediatr. Allergy Immunol..

[24] State Pharmacopoeia Commission (2015). Pharmacopoeia of the People’s Republic of China.

[25] Zhang L., Wang Y., Yang D., Zhang C., Zhang N., Li M., Liu Y. (2015). *Platycodon grandiflorus*–An ethnopharmacological, phytochemical and pharmacological review. J. Ethnopharmacol..

[26] Sun X., Zhang W., Tang P., Jing W., Liu Y., Tang Z. (2017). Detection of Main Components and Antioxidant Activity of *Platycodon grandiflorum* Extracts. Chin. Agric. Sci. Bull..

[27] Sheng Y., Liu G., Wang M., Lv Z., Du P. (2017). A selenium polysaccharide from *Platycodon grandiflorum* rescues PC12 cell death caused by H_2_O_2_ via inhibiting oxidative stress. Int. J. Boil. Macromol..

[28] Xu Y., Dong Q., Qiu H., Cong R., Ding K. (2010). Structural characterization of an arabinogalactan from *Platycodon grandiflorum* roots and antiangiogenic activity of its sulfated derivative. Biomacromolecules.

[29] Zheng P., Fan W., Wang S., Hao P., Wang Y., Wan H., Hao Z., Liu J., Zhao X. (2017). Characterization of polysaccharides extracted from *Platycodon grandiflorus* (Jacq.) A. DC. affecting activation of chicken peritoneal macrophages. Int. J. Boil. Macromol..

[30] Park M.J., Ryu H.S., Kim J.S., Lee H.K., Kang J.S., Yun J., Kim S.Y., Lee M.K., Hong J.T., Kim Y. (2014). *Platycodon grandiflorum* polysaccharide induces dendritic cell maturation via TLR4 signaling. Food Chem. Toxicol..

[31] Liu W., Liu H., Han M. (2013). Polysaccharides from *Platycodon grandiflorum*. Chem. Nat. Compd..

[32] Oka M., Ota N., Mino Y., Iwashita T., Komura H. (1992). Studies on the conformational aspects of inulin oligomers. Chem. Pharm. Bull..

[33] Li J., Zhang X., Cao L., Ji J., Gao J. (2018). Three Inulin-Type Fructans from *Codonopsis pilosula* (Franch.) Nannf. Roots and Their Prebiotic Activity on *Bifidobacterium longum*. Molecules.

[34] Pontes A.G.O., Silva K.L., da Cruz Fonseca S.G., Soares A.A., de Andrade Feitosa J.P., Braz-Filho R., Romero N.R., Bandeira M.A.M. (2016). Identification and determination of the inulin content in the roots of the Northeast Brazilian species *Pombalia calceolaria* L.. Carbohydr. Polym..

[35] Caleffi E.R., Krausová G., Hyršlová I., Paredes L.L.R., dos Santos M.M., Sassaki G.L., Gonçalves R.A.C., de Oliveira A.J.B. (2015). Isolation and prebiotic activity of inulin-type fructan extracted from *Pfaffia glomerata* (Spreng) Pedersen roots. Int. J. Boil. Macromol..

[36] Weitkunat K., Schumann S., Petzke K.J., Blaut M., Loh G., Klaus S. (2015). Effects of dietary inulin on bacterial growth, short-chain fatty acid production and hepatic lipid metabolism in gnotobiotic mice. J. Nutr. Biochem..

[37] Carlson J., Erickson J., Hess J., Gould T., Slavin J. (2017). Prebiotic dietary fiber and gut health: Comparing the in vitro fermentations of beta-glucan, Inulin and Xylooligosaccharide. Nutrients.

[38] Lopes S.M.S., Krausová G., Carneiro J.W.P., Gonçalves J.E., Gonçalves R.A.C., de Oliveira A.J.B. (2017). A new natural source for obtainment of inulin and fructo-oligosaccharides from industrial waste of *Stevia rebaudiana* Bertoni. Food Chem..

[39] De Kivit S., Kraneveld A.D., Garssen J., Willemsen L.E. (2011). Glycan recognition at the interface of the intestinal immune system: Target for immune modulation via dietary components. Eur. J. Pharmacol..

[40] Osorio F., e Sousa C.R. (2011). Myeloid C-type lectin receptors in pathogen recognition and host defense. Immunity.

[41] Elinav E., Strowig T., Henao-Mejia J., Flavell R.A. (2011). Regulation of the antimicrobial response by NLR proteins. Immunity.

[42] Loo Y.-M., Gale M. (2011). Immune signaling by RIG-I-like receptors. Immunity.

[43] Figdor C.G., van Spriel A.B. (2010). Fungal pattern-recognition receptors and tetraspanins: Partners on antigen-presenting cells. Trends Immunol..

[44] Vereyken I.J., Chupin V., Hoekstra F.A., Smeekens S.C., de Kruijff B. (2003). The effect of fructan on membrane lipid organization and dynamics in the dry state. Biophys. J..

[45] Vereyken I.J., Van Kuik J.A., Evers T.H., Rijken P.J., de Kruijff B. (2003). Structural requirements of the fructan-lipid interaction. Biophys. J..

[46] Austarheim I., Christensen B.E., Hegna I.K., Petersen B.O., Duus J.O., Bye R., Michaelsen T.E., Diallo D., Inngjerdingen M., Paulsen B.S. (2012). Chemical and biological characterization of pectin-like polysaccharides from the bark of the Malian medicinal tree *Cola cordifolia*. Carbohydr. Polym..

[47] Zou Y.-F., Chen X.-F., Malterud K.E., Rise F., Barsett H., Inngjerdingen K.T., Michaelsen T.E., Paulsen B.S. (2014). Structural features and complement fixing activity of polysaccharides from *Codonopsis pilosula* Nannf. var. modesta LT Shen roots. Carbohydr. Polym..

[48] Dedonder R. (1952). Carbohydrates of the Jerusalem artichoke. I. Demonstration of a series of glucofructosans in the tubers. Isolation, analysis and structure of the less polymerised members of the series. Bull. Soc. Chim Biol..

[49] Singleton V., Rossi J.A. (1965). Colorimetry of total phenolics with phosphomolybdic-phosphotungstic acid reagents. Am. J. Enol. Vitic..

[50] Bradford M.M. (1976). A rapid and sensitive method for the quantitation of microgram quantities of protein utilizing the principle of protein-dye binding. Anal. Biochem..

[51] Li W., Zhang J., Yu C., Li Q., Dong F., Wang G., Gu G., Guo Z. (2015). Extraction, degree of polymerization determination and prebiotic effect evaluation of inulin from Jerusalem artichoke. Carbohydr. Polym..

[52] Hu J., Cao X., Pang D., Luo Q., Zou Y., Feng B., Li L., Chen Z., Huang C. (2017). Tumor grade related expression of neuroglobin is negatively regulated by PPARγ and confers antioxidant activity in glioma progression. Redox Boil..

